# The role of sense of control and locus of control in depressive and anxious symptoms during COVID-19: an integrative review

**DOI:** 10.1186/s41155-025-00375-w

**Published:** 2026-01-29

**Authors:** Amanda Gabriela Souza Ferreira, Hendrik Wilhelm Crispiniano Garcia, Samily Suelen da Silva, Fábio Antônio Mota Fonseca da Silva, Jonatas Wesley Lira Ferreira, Isvânia Maria Serafim da Silva Lopes

**Affiliations:** 1 Faculdade Pernambucana de Saúde (FPS), Avenida Mal. Mascarenhas de Morais, 4861 - Imbiribeira, Recife, Pernambuco Brazil; 2 Centro Universitário Brasileiro (UNIBRA), Padre Inglês, 257 - Boa Vista, Pernambuco 50050-230 Recife, Brazil; 3 Universidade Federal de Pernambuco (UFPE), Av. Prof. Moraes Rego, 1235 - Cidade Universitária, Pernambuco 50670-901 Recife, Brazil; 4 Centro Universitário Maurício de Nassau (UNINASSAU), R. Guilherme Pinto, 114 - Graças, Pernambuco 52011-210 Recife, Brazil

**Keywords:** Internal–external control, Locus of control, Sense of control, Perceived control, Depression, Anxiety, COVID-19, Mental health

## Abstract

**Background:**

The COVID-19 pandemic triggered a series of psychological impacts, resulting in significant increases in anxiolytic and depressive disorders, influenced by isolation measures, health insecurity, and abrupt social changes. Two key concepts for understanding emotional responses during this period include Sense of Control (SoC) and Locus of Control (LoC). The SoC refers to the general perception of personal agency, while the LoC is a more specific framework that classifies control perceptions as either internal (believing one has control over life events) or external (attributing outcomes to external forces). Previous studies indicate that a more external LoC and a low SoC are associated with greater psychological vulnerability in stressful contexts, such as during the pandemic, which can amplify symptoms of anxiety and depression. This integrative review aims to gather and synthesize studies that explore the relationship between LoC and/or SoC and symptoms of anxiety and depression during the COVID-19 pandemic.

**Main text:**

A systematic search was conducted in PubMed, Scopus, and BVS databases, focusing on studies published within the last 5 years. Quantitative studies with adult samples were considered eligible while excluding those with specific pre-existing conditions. After removing duplicates, titles and abstracts were screened, resulting in 12 full-text studies to be reviewed. Of these, nine met inclusion and exclusion criteria and were included in the analysis. An emerging pattern showed that individuals with external LoC and low SoC had higher levels of anxiety and depression during the COVID-19 pandemic. The role of LoC and SoC as moderators of psychological distress was documented, reinforcing prior evidence that individuals with an external LoC are more vulnerable to mental health challenges in times of crisis. Furthermore, these constructs influenced behavioral responses to the pandemic, with higher SoC and internal LoC associated with more proactive and responsible coping strategies.

**Conclusion:**

Both LoC and SoC play critical roles in understanding the psychological impacts of the pandemic. Despite populational and methodological diversity, most studies point to a clear correlation between perception of control and levels of anxiety and depression, highlighting the importance of interventions that increase the perception of personal control to reduce psychological distress.

## Introduction

The coronavirus pandemic of 2019 (COVID-19) has left profound consequences, which are still being felt in society in 2024 (Bergamo, 2024; Manchia et al., 2022). This has led to an exponential growth in research investigating the impact of the pandemic context on mental health. Although necessary, protective restrictions—such as social isolation, disruptions in work and education, and limitations on social and recreational activities—have contributed to a significant rise in psychological distress (Santomauro et al., 2021).

For example, a meta-analysis review carried out in 2020 revealed that the global prevalence of depression increased from 3.44% in 2017, to 25% in 2020, characterizing a percentage 7 times higher during the pandemic (Bueno-Notivol et al., 2021). This pattern has also been repeated with anxiety disorders, with recent literature from the COVID-19 Mental Disorders Collaborators (Santomauro et al., 2021) pointing to a prevalence of major depressive disorder and anxiety disorders during 2020, both of which were associated with increased rates of SARS-CoV-2 (Severe Acute Respiratory Syndrome Coronavirus 2) infection and decreased human mobility. The data from this research pointed to the addition of 53.2 million new patients with major depressive disorder and 76.2 million cases of new anxiety disorders globally in 2020 (Santomauro et al., 2021).

The psychological burden of the pandemic affected a wide range of individuals, including healthcare workers, students, and workers in different sectors. Healthcare professionals were among the most affected, with prevalence rates of 31.8% for anxiety and 29.4% for depression, with nurses experiencing higher rates than doctors (Maqbali et al., 2024). Among workers from different sectors, uncertainty about financial stability and concerns about personal and family health also led to an increase in these symptoms (Shah et al., 2024). Lastly, university students have also been significantly affected by increased symptoms of depression and anxiety, due to uncertainty about their academic future, social isolation, and the need to adapt to new online learning methods (Deng et al., 2021). 

In the context of stress and uncertainty, the *Sense of Control* (SoC) is a crucial psychological factor that mediates how individuals perceive control over adverse events. SoC refers to control, which involves the belief that it is possible to achieve desired results and avoid or circumvent undesirable ones (Skinner, 1996). SoC is a broad construct that includes various dimensions of perceived control, such as resilience, and the ability to adapt in the face of unpredictable events (Skinner, 1996).

On the other hand, *Locus of Control* (LoC) is a specific aspect of SoC that refers to the perceived origin of this control (Eatough & Spector, 2014). It corresponds to an aspect of personality that indicates a person's inclination to believe that control over events lies in their own actions (internal LoC) or external factors, such as luck or circumstances beyond their reach (external LoC) (Rotter, 1966).

In short, SoC refers to the general and subjective feeling that the individual has control over their life, while LoC is a construct that classifies the perceived origin of control, whether internal or external. Though distinct construct conceptually, due to the scope of SoC, it is commonly used as an umbrella term for various theories involving the perception of control and is therefore commonly used interchangeably with LoC (Abeles, 1991; Rotter, 1966; Skinner, 1996). Despite their sometimes interchangeable use in the literature, results and discussion of both constructs were separated during the production of the article to improve clarity and interpretation of findings.

Several studies have looked at the importance of the perception of control in psychological functioning, especially concerning the development and prevention of psychopathologies (Gallagher et al., 2014; Groth et al., 2019; Schlechter et al., 2023; Sullivan et al., 2017). Studies indicate that individuals with a higher external LoC tend to experience more negative emotions and rely on maladaptive coping strategies such as rumination (Groth et al., 2019).

Consequently, external LoC is strongly associated with increased vulnerability to mental health disorders, particularly depression and anxiety (Benassi et al., 1988; Bliznashki, 2020; Ko & Hsu, 2005). On the other hand, internal LoC serves as a protective factor against psychopathologies. It is generally associated with higher levels of emotional resilience, self-esteem, and more adaptive strategies, such as positive thinking and help-seeking, which leads to lower levels of stress and mental suffering (Rotter, 1966; Ryan & Deci, 2000; Shin & Lee, 2021).

Given its influence on psychological well-being, LoC has received increased attention during the COVID-19 pandemic as researchers sought to understand its relationship with mental health and identify potential areas for clinical intervention. Among such research, the findings indicate that external LoC is directly proportional to mental health problems during COVID-19 (Shin & Lee, 2021). Vulnerable populations, such as women and young adults—groups already identified as being at higher risk for mental illness in the pandemic context (Bareeqa et al., 2021; Bueno-Notivol et al., 2021) – were also found to have a higher external LoC (Shin & Lee, 2021).

In contrast, internal LoC was linked to lower pandemic-related stress (Krampe et al., 2021). More importantly, it was also associated with greater adherence to public health recommendations. Individuals with an internal LoC tend to believe their actions directly impact their health, leading them to engage in protective behaviors such as wearing masks and social distancing (Berg & Lin, 2020). Given that these behaviors can influence community health, the role of LoC extends beyond individual well-being and has public health implications.

Regarding public health, this review is aimed specifically at the "COVID-19 Pandemic era", therefore, for the purposes of this review, the COVID-19 pandemic is defined according to the World Health Organization (WHO) as spanning from March 11, 2020 - when COVID-19 was officially declared a pandemic - until May 5, 2023, when the status of public health emergency of international concern was officially terminated (World Health Organization, 2020).

Although prior research has explored the impact of perceived control on mental health within this defined pandemic period, to date, no integrative review has specifically investigated the relationship between Sense of Control and Locus of Control with depressive and anxious symptoms during this time. Therefore, this integrative review seeks to fill this gap by examining the role of these constructs in the development and maintenance of depression and anxiety throughout the COVID-19 pandemic. 

*Sense of Control and Locus of Control.*

## Methodology

This integrative review was conducted to examine the role of *Sense of Control* (SoC) and *Locus of Control* (LoC) in the development of depressive and anxious symptoms during the COVID-19 pandemic. The methodological framework proposed by Whittemore and Knafl (2005) was followed to systematically synthesize the available evidence, ensuring rigor and comprehensiveness in data collection, analysis, and interpretation. The decision to conduct an integrative review was motivated by the need to capture the complex relationship between *Sense of Control* and/or *Locus of Control* and mental health outcomes, specifically, symptoms of anxiety and depression. Given the broad nature of these constructs—encompassing approximately 100 related terms with overlapping and interchangeable usage (Skinner, 1996)- an intergrative approach allowed for a more comprehensive examination of their various dimensions and interactions across different contexts. In addition, the integrative review covers a variety of populations, including university students, health professionals, and corporate workers, as well as individuals of different nationalities. By incorporating data from multiple demographic groups, this review provides a richer, more contextualized understanding of the psychological impacts of the COVID-19 pandemic, reflecting the diverse experiences and challenges faced by different segments of the population.

### Inclusion and exclusion criteria

Studies included in this review were required to be published within the last five years (2019–2024) in English, Portuguese, or Spanish. However, such studies were only admitted to the selection as long as the collection data was located in the pandemic period defined in this review, according to the definition of the World Health Organization: from March 11, 2020 to May 5, 2023. Eligible studies were conducted within the context of the COVID-19 pandemic and included adult participants aged 18 years or older, regardless of ethnic origin. The review included observational studies, such as cohort, case–control, and cross-sectional designs, as well as clinical trials, whether randomized or non-randomized. To ensure methodological rigor, consistency, and reliability, all studies were required to use validated and standardized instruments to measure Locus of Control (LoC), Sense of Control (SoC), and symptoms of depression and anxiety. The review focused on studies that investigated the relationship between LoC, both internal and external, and depressive and anxious symptoms, with comparisons between different types of LoC (internal versus external) and their effects on these mental health outcomes. Additionally, studies addressing SoC were included due to its conceptual proximity to LoC, broadening the scope of analysis and providing a more comprehensive understanding of how perceptions of control influence mental health during times of crisis. This broader conceptual approach was considered essential to capture the nuanced role of control-related constructs in the context of the pandemic.

Studies were excluded if they met any of the following criteria: 1) adolescent, child, or elderly participants; 2) Severe psychiatric comorbidities or specific clinical conditions that could interfere with the relationship between LoC and depressive/anxious symptoms (e.g. schizophrenia, epilepsy) 3) Any type of reviews.

### Search strategy

The search strategy was based on descriptors related to the LoC and depression and anxiety, with the search operation shown in Table 1. The searches were carried out in the PubMed, Scopus, and Virtual Health Library (BVS) databases. The search results were exported in RIS format and imported into the Rayyan software to facilitate the screening of studies.

**Table 1:** Search operation used

Database	Search operation
Pubmed	(("Internal–External Control"[Mesh] OR "Locus of Control" OR "Control Locus") AND ("Depression" OR "Depressive Disorder" OR "Anxiety" OR "Anxiety Disorders"[Mesh])) NOT ("Review"[Publication Type] OR "Systematic Review"[Publication Type] OR "Meta-Analysis" [Publication Type])
Scopus	(("Internal–External Control" OR "Locus of Control" OR "Control Locus") AND ("Depression" OR "Depressive Disorder" OR "Anxiety" OR "Anxiety Disorders"))
BVS	("Internal–External Control" OR "Locus of Control" OR "Control Locus") AND ("Depression" OR "Depressive Disorder" OR "Anxiety" OR "Anxiety Disorders")

The search strategy involved querying multiple databases using a combination of keywords and subject headings related to locus of control and mental health conditions. In PubMed, the search was conducted using the terms ("Internal–External Control"[Mesh] OR "Locus of Control" OR "Control Locus") AND ("Depression" OR "Depressive Disorder" OR "Anxiety" OR "Anxiety Disorders"[Mesh]), while excluding reviews, systematic reviews, and meta-analyses by applying the NOT operator with ("Review"[Publication Type] OR "Systematic Review"[Publication Type] OR "Meta-Analysis" [Publication Type]). For Scopus, a similar strategy was used, incorporating ("Internal–External Control" OR "Locus of Control" OR "Control Locus") AND ("Depression" OR "Depressive Disorder" OR "Anxiety" OR "Anxiety Disorders"). The search in BVS followed the same structure, using ("Internal–External Control" OR "Locus of Control" OR "Control Locus") AND ("Depression" OR "Depressive Disorder" OR "Anxiety" OR "Anxiety Disorders"). This approach ensured a comprehensive retrieval of relevant studies across different databases while refining results to exclude review articles in PubMed.

### Study selection process

**Fig. 1:**
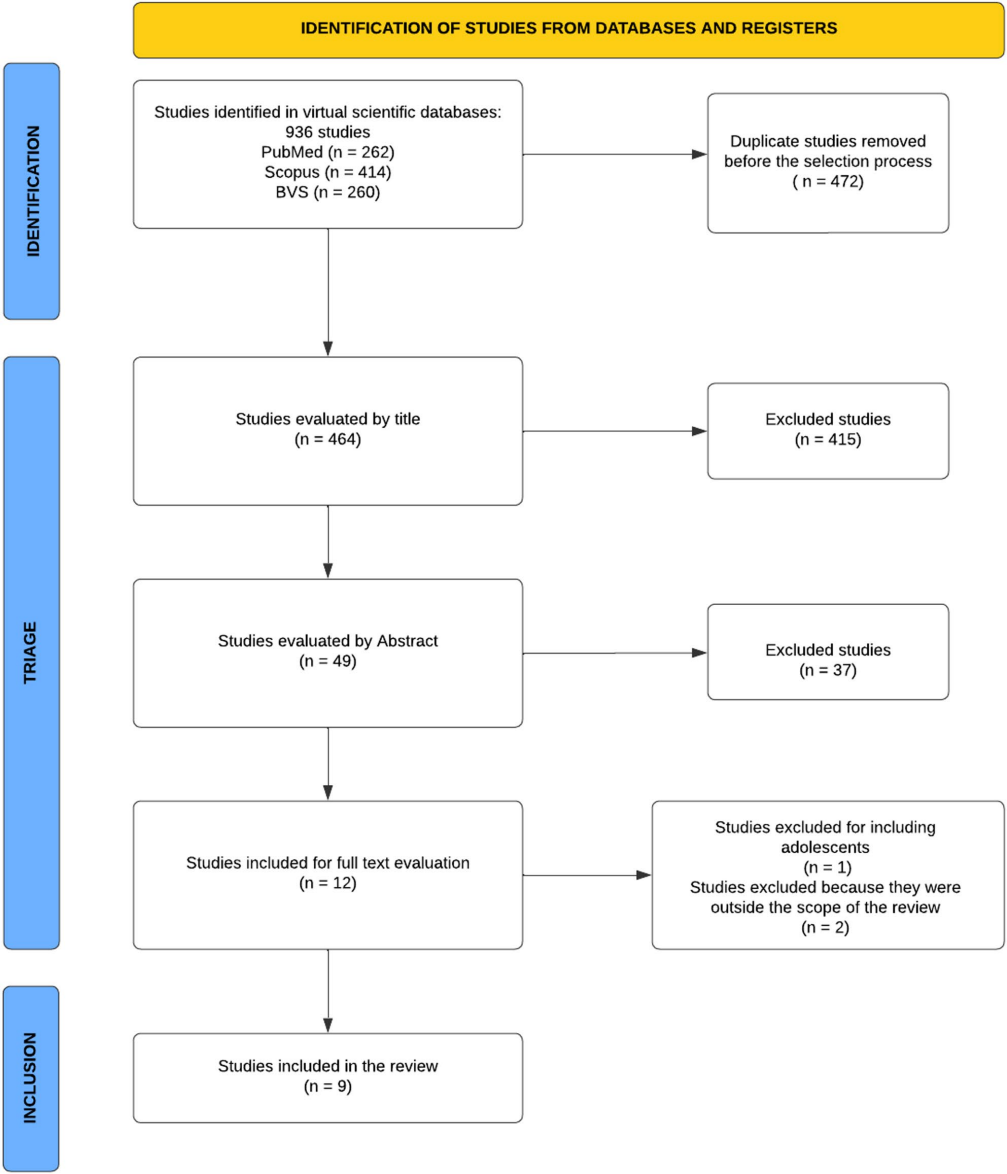
PRISMA 2020 flowchart of the selection process for studies included in the review

The study selection process followed a systematic approach to ensure accuracy and consistency. Initially, 936 studies were retrieved from three databases: PubMed, Scopus, and Virtual Health Library (BVS). After importing the results into Rayyan, duplicates were manually identified and removed to ensure no repeated studies remained in the sample. Of the 936 articles identified, 473 duplicates were removed, resulting in 463 unique studies for title and abstract screening. The study selection was conducted in three stages: (1) initial screening by title, (2) abstract screening, and (3) full-text review. Two independent reviewers (Including the initials of researchers) conducted the screening process in a blinded manner, and any discrepancies were resolved through consensus.Of the 463 studies, 49 were selected based on their titles. After reviewing the abstracts, 12 studies met the inclusion criteria and proceeded to full-text analysis. Following an in-depth discussion between reviewers, 9 studies were ultimately included in this review (Fig. 1).

### Data extraction

Data extraction was carried out following a pre-defined protocol to ensure the accuracy and coherence of the information collected. The process was carried out manually and systematically, using a standardized spreadsheet specifically designed for this review. This spreadsheet included key variables of interest, ensuring relevance to the study’s scope. The information extracted included the full name of the study, authors, study design, population characteristics (such as sample size, age range, and inclusion/exclusion criteria), type of locus of control investigated (internal, external, or health) or whether the study assessed SoC, as well as the type of anxiety and depression assessed, including specifications on the nature of the symptoms, if any.

In addition, the main objectives of each study, the instruments used to measure *LoC* and SoC, and anxiety symptoms were recorded. The main conclusions and results of the studies were extracted and entered into the spreadsheet. Finally, the methodological limitations of each study were also recorded, as reported by the authors, to assess the robustness of the evidence presented.

### Data synthesis and analysis

The studies included in the review were analyzed qualitatively, to identify patterns and relationships between different types of Locus of Control (LoC) and Sense of Control (SoC) and their association with depressive and anxious symptoms.. The direct and indirect impacts of the COVID-19 pandemic on these constructs were considered, as well as how these factors influenced different population groups.

### Methodological quality of studies

The methodological quality of the studies included in this review was analyzed using the CASP (Critical Appraisal Skills Programme) checklist, applying the version for cross-sectional studies to descriptive designs and the version for cohort studies to longitudinal designs. A total of nine studies were evaluated, six cross-sectional (Wu et al., 2024; Sigurvinsdottir et al., 2020; Krampe et al., 2021; Mahmoud et al., 2022; Maršanić et al., 2023; Vally et al., 2023) and three longitudinal (Msetfi et al., 2022; Eren et al., 2023; Guttmann-Ducke et al., 2023).All the studies addressed clearly defined questions and used appropriate methods to answer their research questions. However, variation was observed in the quality of recruitment. Among the cross-sectional studies, Wu et al., Sigurvinsdottir et al. and Krampe et al. presented recruitment processes considered adequate, while Mahmoud et al., Maršanić et al. and Vally et al. used strategies susceptible to selection bias, such as convenience or snowball sampling, which can compromise the representativeness of the samples. Among the longitudinal studies, only Eren et al. showed consistent recruitment and follow-up; the others suffered significant sample losses and limitations in maintaining the cohort. In particular, the study by Msetfi et al. had a retention rate of only 15% between the two collection phases, as well as a relatively short interval between the evaluation moments (approximately four months), which limits its ability to capture significant changes over time and compromises the robustness of the longitudinal follow-up.All the studies used validated instruments and reliable measurements to minimize bias, and the data analysis was conducted properly. The presentations of the results were clear and aligned with the research questions. However, none of the studies evaluated discussed in depth the applicability of their findings to the local population, which limits the generalizability of the results.Overall, the studies included had satisfactory methodological quality and contributed relevant findings to understanding the effects of locus of control and sense of control on depressive and anxiety symptoms during the pandemic. Even so, there is a need for greater attention to be paid to sample representativeness, minimizing loss to follow-up and the adequate duration of longitudinal follow-up in future studies. The results of the analysis can be seen in Tables 2 and 3.

**Table 2:** CASP Checklist: For Descriptive/Cross-Sectional Studies

Studies	Wu, et al.,(2024)	Sigurvinsdottir, et al., (2020)	Krampe, et al., (2021)	Mahmoud, et al., (2022)	Maršanić, V. et al. (2023)	Vally, et al., (2023)
1. Did the study address a clearly focused issue?	Yes	Yes	Yes	Yes	Yes	Yes
2. Did the authors use an appropriate method to answer their question?	Yes	Yes	Yes	Yes	Yes	Yes
3. Were the subjects recruited in an acceptable way?	Yes	Yes	Yes	No	No	No
4. Were the measures accurately measured to reduce bias?	Yes	Yes	Yes	Yes	Yes	Yes
5. Were the data collected in a way that addressed the research issue?	Yes	Yes	Yes	Yes	Yes	Yes
6. Did the study have enough participants to minimise the play of chance?	Yes	Yes	Yes	Yes	Yes	Yes
7. How are the results presented and what is the main result?	Yes	Yes	Yes	Yes	Yes	Yes
8. Was the data analysis sufficiently rigorous?	Yes	Yes	Yes	Yes	Yes	Yes
9. Is there a clear statement of findings?	Yes	Yes	Yes	Yes	Yes	Yes
10. Can the results be applied to the local Population?	Cant´t tell	Cant´t tell	Cant´t tell	Cant´t tell	Cant´t tell	Cant´t tell
11. How valuable is the research?	Yes	Yes	Yes	Yes	Yes	Yes

**Table 3:** CASP Checklist: For Cohort Studies

Studies	Msetfi, R. et al. (2022)	Eren, et al., (2023)	Guttmann-Ducke, et al., (2023)
1. Did the study address a clearly focused issue?	Yes	Yes	Yes
2. Was the cohort recruited in an acceptable way?	No	Yes	No
3. Was the exposure accurately measured to minimise bias?	Yes	Yes	Yes
4. Was the outcome accurately measured to minimise bias?	Yes	Yes	Yes
5. Have the authors identified all important confounding factors?	Can't tell	Yes	Can't tell
b) Have they taken account of the confounding factors in the design and/or analysis?	Yes	Yes	Yes
6. a) Was the follow-up of subjects complete enough?	No	Yes	No
b) Was the follow-up of subjects long enough?	No	Yes	Yes
7. What are the results of this study?	Yes	Yes	Yes
8. How precise are the results?	Yes	Yes	Yes
9. Do you believe the results?	Can't tell	Yes	Yes
10. Can the results be applied to the local population?	Can't tell	Can't tell	Can't tell
11. Do the results of this study fit with other available evidence?	Yes	Yes	Yes
12. What are the implications of this study for practice?	Yes	Yes	Yes

## Results

This integrative review analyzed a total of nine studies, three of which focused on the Sense of Control (SoC) in the context of the COVID-19 pandemic, while six investigated Locus of Control (LoC) in that same context. The following sections detail the key findings and methodological aspects of these studies. Tables 4, 5 and 6, used at the end of each session, show details of the studies, such as the number of participants, the location of the study, etc. However, in the section on the instruments used, only the directly relevant to the focus of this research have been reported.

**Table 4:** Synthesis of study data on SoC

Studies	Vally, et al., (2023)	Wu, et al., (2024)	Msetfi, R. et al. (2022)
Study objective	To examine whether variation in SoC explained the relationship between depression and social media addiction during the pandemic	To examine the indirect roles of SoC and intolerance of uncertainty between pandemic fatigue and depressive symptoms	To explore the relationship between public health restrictions, SoC, and depression
Type of Study	Descriptive/Cross-Sectional	Descriptive/Cross-Sectional	Cohort
Location	Egypt and UAE	China	Ireland
Sample size	1322 participants (997 female)	1162 participants (747 female)	314 participants in the first period (192 female), and 47 in the second
Instruments	DASS-21 scale, Brailovskaia and Margraf 2-item Scale for SoC, BSMAS Scale	Pandemic Fatigue scale, Personal Domain Scale, and Depression Subscale of the DASS-21	BDI Scale, Sense of Control Scale, PHR questionnaire with Scale,
Main results	SoC was negatively associated with depressive symptoms and social media addiction, mediating this relationship	SoC acted as a protective factor against pandemic fatigue, intolerance to uncertainty, and depressive symptoms	Low SoC was associated with a greater likelihood of depressive symptoms. High SoC was correlated with a lower impact of restrictions and a lower probability of depression

*DASS-21* Depression, Anxiety and Stress Scales, *BSMAS* Bergen Social Media Addiction Scale, *BDI* Beck Depression Inventory, *PHR* Experience of Public Health Restrictions

**Table 5:** Synthesis of study data on LoC—Descriptive/Cross-Sectional studies

Studies	Sigurvinsdottir, et al., (2020)	Mahmoud, et al., (2022)	Krampe, et al., (2021)	Maršanić, V. et al. (2023)
Study objective	To examine the relationship between LoC, depressive and anxious symptoms, and internet use during the pandemic	To analyze the moderating role of the LoC in the organizational context and its psychological impacts during the pandemic	To investigate whether the LoC moderated the relationship between COVID-19 stress and mental distress in the general population	To investigate the relationship between the COVID-19 pandemic, mental health, and its association with perceptions of the pandemic and locus of control
Location	USA	Middle East and North Africa	Norway and Germany	Croatia
Sample size	1723 participants (798 female)	847 participants (364 female)	1225 Norwegians (897 female) and 1,527 Germans (993 female)	1482 participants (1230 female)
Instruments	DASS-21 Scale, ISCS e Levenson's LoC Scale	HAM-A Scale, WLCS Scale, Work Alienation Scale, Job Insecurity Scale	LoC-4 Scale, COVID-19 Stress Scale, PHQ-4 Questionnaire	DASS-21 Scale, Rotter's LoC Scale
Main results	LoC moderated the relationship between social capital and anxiety. People with external LoC had a higher risk of depressive and anxious symptoms	The external locus of control increases vulnerability to alienation at work, as it intensifies the impact of anxiety on the perception of lack of control and disconnection in the context of insecurity	The external locus of control intensifies the relationship between the stress caused by COVID-19 and symptoms of anxiety and depression, while the internal locus of control acts as a protective factor.	The external locus of control has been shown to be associated with higher levels of anxiety, stress and depression, indicating that individuals with this orientation are more vulnerable to these symptoms

*DASS- 21* Depression, Anxiety and Stress Scales, *ISCS* Internet Social Capital Scale, HAM-A; the Hamilton Anxiety Rating Scale, *WLCS* Work Locus of Control Scale, *PHQ-4* Patient Health Questionnaire

**Table 6:** Synthesis of study data on LoC – Cohort studies

Studies	Eren, et al., (2023)	Guttmann-Ducke, et al., (2023)
Study objective	To assess personality traits and mental distress in young French adults during the pandemic	To investigate the association between LoC and mental health during the pandemic, with a focus on the development of PTSD
Location	France	Austria
Sample size	904 participants	914 participants
Instruments	Anxious/Depressed scale from the ASEBA questionnaire, ICI Scale	FKK Questionnaire, DASS Scale and Short Screening Scale for PTSD
Main results	Internal LoC reduced the risk of anxiety/depression symptoms by 30% for every 10-point increase. External LoC is associated with higher mental health risk	External LoC and low self-confidence are associated with a higher risk of PTSD, depression, anxiety, and stress

*ASEBA* Achenbach System of Empirically Based Assessment, *ICI* Internal Control Index, *FKK* Questionnaire on Competence and Control Beliefs, *DASS* Depression-Anxiety and Stress-Score

### SENSE OF CONTROL (SoC)

On the side of SoC, studies analysed whereby Vally, et al., (2023), Wu, et al., (2024) and Msetfi, R. et al. (2022) on the *Sense of Control* in the pandemic context. Two of these employed a cross-sectional design, while one was longitudinal, offering both static and dynamic perspectives on SoC in different populations. These studies were conducted in diverse geographical contexts, including the United Arab Emirates (UAE), China, and Ireland respectively, contributing to a broader understanding of SoC in pandemic-related mental health.

The study by Vally, et al. 2023) examined whether variance *in Sense of Control* explained the association between depression and social media addiction, with university-age students enrolled in two universities in Egypt and the UAE. Data was collected through convenience sampling. The main results of this study were that SoC was negatively associated with depressive symptoms and social media addiction during the pandemic, significantly mediating the relationship between these variables.

The study by Wu, et al., (2024) covered other factors and aimed to examine the indirect roles of a sense of control and intolerance of uncertainty in the association between pandemic fatigue and depressive symptoms. Chinese university students aged 18 took part in the study. The main results of this study point to the SoC as a protective factor against pandemic fatigue, intolerance of uncertainty, and depressive symptoms. Students who experienced a decrease in SoC due to pandemic fatigue were less able to tolerate uncertainty.

The third study, Msetfi, R. et al. (2022), sought to explore the relationship between the experience of public health restrictions, a sense of control, and depression. The study's data collection was divided into two periods, the first covering the period between January 7 and February 22, 2022, and the second being limited to April 1 and May 17, 2022. It is worth noting that in the first period, there were stricter public health restrictions in place, while in the second period, there were no public health restrictions in place in Ireland. In the first period, the sample included 314 participants, while in the second there was a sharp decrease, leaving 47 participants.. The main findings of this study showed that the likelihood of experiencing depressive symptoms was higher for those with low SoC. Those with lower levels of depression had high levels of SoC, and high SoC was correlated with low impact of restrictions and were less likely to show signs of depression.

The three studies had a relatively large sample size, a well-defined methodology, and results that responded to the stipulated objective. All the studies in this category, despite having different directions within the same context, have similar outcomes, which point to high levels of sense of control as a protective factor against depressive symptoms during the COVID-19 pandemic.

### Locus of control

This integrative review analysed six studies that investigated *Locus of Control* (LoC) in different contexts. Of these, four had a cross-sectional design, while two were prospective longitudinal studies. The distinction between these two types of studies is relevant, as each approach provides different perspectives on behavior and its changes over time. Cross-sectional studies offer a static view, while longitudinal studies capture temporal dynamics.

The cross-sectional studies included samples from various regions, such as the Middle East, North Africa, Croatia, Norwa,y, and the United States, offering a diverse cultural and socio-economic perspective on LoC.

The study by Sigurvinsdottir, et al., (2020), conducted in the United States and investigated the relationship between LoC and depressive and anxiety symptoms, focusing on the impact of LoC on Internet use In this study, it was found that the LoC acted as a moderator between social capital and anxiety. In other words, the impact of social capital on anxiety depends on the person's type of LoC (internal or external). Initially, a positive correlation was observed between internet social capital and anxiety levels, indicating that online interactions might be linked to increased anxiety. However, after accounting for the Locus of Control, this relationship was no longer present. In addition, this study also found that during the pandemic, the LoC proved to be essential to mental health, in which individuals with external LoC were observed to be more likely to develop symptoms of depression and anxiety.

The study by Mahmoud, et al., (2022), carried out with employees of a hotel company in the Middle East and North Africa, examined the moderating role of LoC in the organizational context, assessing the psychological impacts of the pandemic on the work environment. The results indicate that the LoC affects the way anxiety is linked to alienation. In this case, it appears that employees with a LoC are more vulnerable to moving from anxiety to alienation, due to job insecurity resulting from the effects of COVID-19.

Krampe et al. (2021) studied 1,225 Norwegians and 1,527 Germans, while Maršanić et al. (2023) examined 1,482 Croatian participants, both investigating Locus of Control (LoC) in the general adult population without contextual restrictions. The analysis of these two studies revealed that internal LoC acted as a protective factor: as internal LoC scores increased, the impact of COVID-19 stress on mental distress decreased. On the other hand, external LoC exacerbated the negative effects of the pandemic, being associated with drastically worse mental health.

Eren, et al., (2023) longitudinal study assessed personality traits and mental distress following the COVID-19 test. The study design allowed for the continuous follow-up of a specific cohort of young French adults over several years. The study participants are the children of individuals belonging to the GAZEL cohort, a sample of French workers initially recruited in 1989. Data for the study began to be collected in 2009, with subsequent collections carried out in 2011, 2015, and 2018. These collections were made utilizing self-administered questionnaires, sent periodically to the participants, to investigate aspects related to mental health, risk behaviours and social and psychological factors.

With the onset of the COVID-19 pandemic, the study incorporated a new phase of data collection to assess the impacts of the pandemic on the mental health and addictive behaviors of young adults. Between March 2020 and May 2021, nine waves of online questionnaires were carried out with 904 adult participants. The first five waves were administered weekly, followed by two fortnightly waves and two additional waves, carried out in June-July 2020 and between December 2020 and May 2021. This short interval collection format aimed to capture immediate changes and the evolution of psychological and behavioral effects as the pandemic progressed.

The questionnaires covered a wide range of topics, including mental health measures such as symptoms of depression, anxiety and stress, and risk behaviors such as substance use and internet addiction. Additionally, sociodemographic and psychological variables, including the LoC, were included to assess possible moderating or protective factors against the impacts of the pandemic.

This study also revealed that the external LoC was a significant risk factor for increased symptoms of anxiety and depression. The results showed that for every 10-point increase in internal LoC, the chances of manifesting clinically significant symptoms of anxiety and/or depression fell by 30%, confirming that internal LoC can have a protective effect. Therefore, individuals with a more external LoC are at greater risk of experiencing symptoms of anxiety and/or depression than those with a more internal LoC. In addition, the feeling of lack of control over the virus and the restrictions imposed can intensify the feeling of powerlessness in individuals with external LoC, which may in turn increase the risk of mental health problems.

The second longitudinal study, by Guttmann-Ducke, et al., (2023), was carried out in Austria and focused on the association between individuals' LoC and mental health during the pandemic. This study initially included 1,556 individuals, but only 914 participants completed all stages of the research. The methodology of this study was organized into four separate visits, to track changes in participants' mental health status over a year, in response to the COVID-19 pandemic.

Between March and December 2021, online versions of multiple psychological and health-related questionnaires were administered to assess participants' mental health status, including symptoms of post-traumatic stress. This initial assessment provided a comprehensive baseline for evaluating psychological distress during the pandemic.

A follow-up assessment was conducted 48 h after participants received a negative COVID-19 test result to examine potential relief effects on psychological stress. This measurement aimed to capture the immediate emotional impact of receiving a negative diagnosis within the context of pandemic-related anxiety.

Ninety days after the initial assessment, a third evaluation was carried out to monitor changes in depression, anxiety, stress, and post-traumatic stress symptoms over time. This interval was chosen to identify potential gradual shifts in psychological distress. Finally, a fourth assessment, conducted nine months after the first, focused on the persistence of post-traumatic stress symptoms, offering insights into the long-term psychological effects of the pandemic.

The findings indicated that participants who screened positive for post-traumatic stress tended to exhibit an external locus of control, reflecting a diminished sense of personal agency over their circumstances. Lower self-efficacy and reduced internal control were associated with a decreased likelihood of developing post-traumatic stress symptoms, whereas a stronger reliance on external factors emerged as a risk factor.

Additionally, participants with post-traumatic stress symptoms consistently reported higher levels of depression, anxiety, and stress throughout the study. These symptoms were linked to a heightened sense of helplessness, exacerbated by external influences such as media exposure and restrictive governmental measures. The combination of an external locus of control and low self-confidence was particularly associated with increased psychological distress, reinforcing its role in the development and persistence of post-traumatic stress during the pandemic.

## Discussion

### SENSE OF CONTROL (SoC)

This research aimed to investigate, through an integrative review, how LoC (internal or external) and SoC influenced the development and severity of anxiety and depression symptoms during the COVID-19 pandemic. SoC is an adaptive and motivational variable that refers to the belief that it is possible to influence your outcomes through your own efforts. It is an important personal resource, helping to improve well-being and alleviate psychological suffering (Hong et al., 2021).

The relevance of SoC became especially evident in the context of the COVID-19 pandemic, which was marked by widespread pandemic fatigue. Key predictors of pandemic fatigue included isolation, the need to attend work in person, infection of friends and family, and the negative perception of media reports on the pandemic (Ferreira et al., 2022). In response to these challenges, Wu et al. (2024) found that Sense of Control functioned as a protective factor against pandemic fatigue in university students, intolerance to uncertainty and depressive symptoms, being significant in maintaining well-being and reducing psychological suffering (Bueno-Notivol et al., 2021). Supporting this finding, external studies point out that SoC also played a role in the adoption of pro-health behaviors, consequently influencing better physical health outcomes (Hong et al., 2021). Lastly, it's relevant to point out, that in Wu et al. (2024) sample of 1,162 Chinese students, 64.29% were female, reflecting a pattern seen across multiple studies where women comprised the majority of participants, potentially influencing the generalizability of SoC findings.

The pandemic and social isolation significantly impacted the health of university students, who, faced with the transition to remote education, were surrounded by uncertainty and fear. Students from lower socioeconomic backgrounds faced an additional challenge, without access to the necessary technology or quality internet (Sunde et al., 2022). In this context, the study by Zahir Vally, Mai Helmy & Louis Fourie (2021), carried out with 1,322 students from universities in Egypt and the UAE, found that during the pandemic, Sense of Control also played a protective role for this population, being negatively associated with depression and addiction to social media, significantly mediating the relationship between these two variables. However, the high proportion of female participants (75.4%) in this study raises important questions about potential gender-specific pathways in how SoC mediates the relationship between depression and social media addiction.

Furthermore, students with low levels of Sense of Control concerning the pandemic experience were more likely to acquire addictions to social media, thus affecting their psychological well-being. Restrictions during the pandemic have led to an increase in smartphone use, especially among university students, children, and adolescents. Consistent with the findings in the previous study, a study outside the sample of this review showed that the increase in screen use during the pandemic had a direct link with increased levels of stress, depression, and anxiety (Santos et al., 2023). However, despite the adverse effects, the use of smartphones in a balanced way has also brought benefits, helping people cope with social distancing and maintain mental well-being (Filho et al., 2023).

Among the general population, the study by Msetfi et al. (2022) showed that during the COVID-19 pandemic, subjects with a low level of Sense of Control were more likely to be categorized as depressed. With 61% of female participants, this study's findings align with broader patterns showing gender differences in control perception during crises, though the inverse relationship between sense of control and depression remained robust across both ganderFurthermore, those with the lowest levels of depression had the highest levels of Sense of Control, pointing to an inversely proportional relationship between symptoms of depression and levels of Sense of Control. This study found that during the pandemic, a Sense of Control had a protective effect, especially on the perception of external restrictions. In line with this, external studies reinforce these findings. Supporting these findings, a systematic review and meta-analysis published in 2024, which analysed 20 articles, showed that all but one of the studies reported a significant relationship between SoC and depressive symptoms. Higher levels of SoC are inversely proportional to higher levels of depression, so the higher the SoC, the lower the level of depression (Msetfi et al., 2024).

When examining SoC across different populations, a clear pattern emerges: university students consistently showed the strongest mediating effects of SoC on mental health outcomes, possibly due to their developmental stage where control beliefs are still forming and more malleable. The general population showed more stable but still significant SoC effects, suggesting that while SoC remains protective across age groups, its malleability and therefore its potential as an intervention target may be highest in younger populations. Yet, caution is warranted regarding this interpretation, as it might also stem from the fact that out of the 3 included studies on SoC, 2 of them were of younger population, particularly, university students.

In conclusion, this review highlights the importance of Sense of Control (SoC) as a protective factor against negative psychological effects during the COVID-19 pandemic. By offering a perception of control over one's own actions and outcomes, SoC is effective in mitigating symptoms of depression, anxiety, and pandemic fatigue, promoting well-being in a context of uncertainty. Therefore, findings indicate the potential of SoC as a promising target for psychological interventions in times of crisis, highlighting the importance of encouraging SoC within educational, organizational, and public health contexts due to its benefits for well-being and psychological health.

### LOCUS OF CONTROL (LoC)

The findings of this study corroborate the existing literature on the role of LoC in mediating psychological impacts in crises, such as the COVID-19 pandemic (Berg & Lin, 2020; Shin & Lee, 2021). Studies by Krampe et al. (2021), Maršanić et al. (2023) and Eren et al. (2023) show that individuals with an internal LoC experienced a lower impact of the pandemic on their mental health, which suggests more adaptive responses and increased resilience in the face of the uncertainties and challenges imposed by the pandemic context. The finding that internal LoC acts as a protective factor against psychological distress reinforces the understanding that individuals who perceive greater control over their lives tend to adopt more effective coping strategies, thus reducing the negative effects of stress (Groth et al., 2019; Rotter, 1966).

On the other hand, these studies have also shown an association between the external LoC and increased symptoms of anxiety and depression, which highlights the vulnerability of individuals who perceive themselves as victims of external forces (Schlechter et al., 2023; Sullivan et al., 2017). These individuals, who tend to attribute control of events to factors outside their influence, show less ability to manage stressors and are more susceptible to psychological distress when faced with situations beyond their control (Rotter, 1966). The feeling of powerlessness with the spread of the virus and social restrictions reinforces this vulnerability, as individuals with external LoC face greater difficulties in dealing with isolation, lack of predictability, and forced changes (Everest, 2024; Rotter, 1966). This aspect becomes even more relevant when considering the particularities of the pandemic, a prolonged event with a high level of uncertainty, which exposes the fragility of those who do not feel they have control over the environment around them (Mahat-Shamir et al., 2023). As such, studies analysed highlight the moderating function of LoC in various psychological and behavioral relationships, broadening the understanding of how different types of LoC can affect not only the levels of depression and anxiety symptoms but also the way these symptoms interact with other psychological factors.

In the study by Sigurvinsdottir et al. (2020), LoC emerges as a moderator in the relationship between Internet social capital and anxiety. Initially, a positive correlation was observed between Internet social capital and anxiety levels, indicating that online interactions could contribute to increased anxiety. However, when the LoC was included in the analysis, this relationship disappeared, suggesting that people's perception of control influences how Internet social capital affects anxiety. For individuals with internal LoC, who perceive themselves as more able to control their reactions and circumstances, online interactions can be seen as less threatening or stressful. In contrast, those with external LoC, who feel that their lives are guided by external factors, may experience online interactions in a more anxious way, attributing virtual interactions a greater influence on their emotional well-being.

These findings are particularly relevant when considering the evidence on the influence of LoC on problematic internet use. Research indicates that internet addiction tends to be negatively associated with internal LoC and positively associated with external LoC (Chak & Leung, 2004). Thus, external LoC not only intensifies anxiety in virtual interactions but can also aggravate digital addiction behaviors. This effect is especially worrying in the context of the pandemic which has led many people to increase their use of social media as a way of adapting (Masaeli & Farhadi, 2021), but, may have intensified the negative impact for those with an external perception of control, increasing the risk of emotional problems and digital addiction.

The study by Mahmoud et al. (2022) also highlights the LoC as a moderator in the transition from anxiety to alienation in contexts of job insecurity. Alienation, in this context, is the feeling of isolation and helplessness that arises when the employee feels a lack of purpose or connection with their work, especially in situations of insecurity exacerbated by the pandemic (Banai and Reisel, (2007). Individuals with external LoC, who feel less control over decisions and events in their lives, are more vulnerable to moving from initial anxiety to alienation, as they have fewer internal resources to manage the sense of uncertainty at work. These findings indicate that, in addition to intensifying anxiety, job insecurity can promote feelings of alienation, particularly in individuals with external LOC, increasing their risk of isolation and emotional disconnection in the work environment.

The relationship between LoC and alienation was explored in another study which shows that external LoC tends to be associated with higher levels of alienation, while internal LoC can attenuate this feeling (de Man & Devisse, 1987). In it, they investigated this connection among university students and found that alienation was significantly associated with an external LoC and low self-esteem. These findings indicate that for individuals with an external LoC, there is a greater tendency to feel powerless and socially disconnected, especially in contexts of uncertainty and lack of control (de Man & Devisse, 1987).

In the workplace, the literature shows that internal LoC is associated with lower levels of emotional distress, job stress, intention to quit, and job turnover (Anderson et al., 2016). It has also been shown to be associated with job satisfaction, motivation, and performance, as well as career success and the use of coping strategies (Anderson et al., 2016). During the pandemic, LoC has become even more relevant, as many workers face additional challenges due to economic uncertainty and health risks (Shah et al., 2024). This highlights the importance of internal LoC as a protective factor against anxiety and alienation in times of crisis.

Finally, Guttmann-Ducke et al. (2023) show a significant association between external LoC and the presence of PTSD symptoms during the pandemic, with individuals with external LoC also exhibiting high levels of depression, anxiety, and stress. This finding suggests that individuals with PTSD and an external LOC profile experience a limited perception of control, which intensifies the emotional reaction to stress and makes coping more difficult. Research corroborates these findings. One study indicated that externalizing control beliefs in the workplace were associated with more PTSD symptoms in firefighters (Onyedire et al., 2017). In addition, another study of 262 Israeli soldiers showed a significant relationship between LoC and PTSD (Solomon et al., 1988), reinforcing the findings obtained by Guttmann-Ducke et al. (2023) in the pandemic context.

The fact that the external LoC is a risk factor for developing PTSD makes it a relevant focus for interventions during the pandemic, especially considering the significant increase in PTSD cases during COVID-19 (Chamaa et al., 2021; Yunitri et al., 2022). Prolonged quarantine can cause psychological distress due to factors such as unemployment, financial and personal losses, constant exposure to media and fake news, fear of death, switching to online education, domestic violence, and the fear of contamination (Chamaa et al., 2021). These stressors have not only contributed to the increase in PTSD cases but have also significantly increased symptoms of depression and anxiety in healthy individuals (Chamaa et al., 2021).

Regarding gendered differences, a consistent pattern emerged across the reviewed studies. Women comprised the majority of participants and demonstrated both higher rates of external LoC and greater vulnerability to anxiety and depression during the pandemic. This gender imbalance was remarkably consistent across different cultural contexts: Krampe et al. (2021) reported 73.2% female participants in their Norwegian sample and 65% in their German sample; Maršanić et al. (2023) found 83% female participants in Croatia; and Eren et al. (2023) documented 66.2% female participants in France. The mental health disparities associated with these gender differences were substantial, with Eren et al. (2023) finding that females had an odds ratio of 1.85 (95% CI [1.31-2.63]) for experiencing clinically significant anxiety and depression symptoms compared to males, while simultaneously demonstrating more external LoC orientations. Similarly, Maršanić et al. (2023) reported significantly higher anxiety (t=-2.99, p<.05) and stress (t=-4.17, p<.01) scores among women. These findings align with the broader literature on affective and internalizing disorders, where women consistently demonstrate greater vulnerability to anxiety and depression, (Farhane-Medina et al., 2022; Nolen-Hoeksema, 2012) suggesting that the pandemic may have amplified pre-existing gender-based mental health disparities through mechanisms involving perceived control.

The relationship between gender and LoC was further nuanced by contextual factors including age and occupational setting. Krampe et al. (2021) found that in their Norwegian sample (mean age 50.26 years) versus their German-speaking sample (mean age 40.35 years), older women with internal LoC showed better mental health outcomes than younger women, suggesting that life experience may strengthen the protective effects of internal LoC or that developmental factors moderate the LoC-mental health relationship. Interestingly, studies with different gender compositions still demonstrated robust LoC effects: Sigurvinsdottir et al. (2020) had a more balanced gender distribution (46.3% female) yet found significant moderating effects of LoC on the relationship between Internet social capital and anxiety, while a study of hospitality workers showed a reversed gender pattern with 57% male participants but maintained strong LoC moderation effects, with external LoC employees showing stronger relationships between job insecurity and anxiety. This suggests that while gender may influence the magnitude of LoC effects, the fundamental relationships between control beliefs and mental health remain consistent across gender compositions and may be particularly influenced by occupational and environmental contexts.

The intersection of gender with LoC and pandemic experiences reveals a complex vulnerability profile that extends beyond simple gender differences. Women's higher rates of external LoC during the pandemic likely reflect not only individual psychological orientations but also structural realities including increased caregiving responsibilities, higher representation in high-exposure service sectors, elevated domestic violence risks, and employment instability. When combined with direct pandemic exposure, this creates particularly vulnerable profiles, as evidenced in studies where 60.8% of female participants tested for COVID-19 showed heightened risk for severe psychological symptoms including PTSD when external LoC was present. 

In summary, the findings of this study reinforce the crucial role of Locus of Control (LoC) in mediating psychological impacts in crisis contexts, such as the COVID-19 pandemic. These associations highlight that the perception of control not only influences how individuals react to external challenges but also affects the use of internal resources to deal with adversity.Importantly, the consistent gender differences observed across studies - with women demonstrating both higher rates of external LoC and greater vulnerability to anxiety and depression - indicate that effective psychological interventions must go beyond simply strengthening internal LoC at the individual level. Interventions should adopt gender-sensitive approaches that acknowledge how differences in caregiving responsibilities, occupational vulnerabilities, differential exposure to pandemic stressors shape individuals' sense of control over their lives. By addressing both the cognitive aspects of control beliefs and the social conditions that constrain agency in an individualized way, interventions can more effectively promote mental health resilience and reduce the psychological impact of prolonged crises in future scenarios. 

### INTEGRATIVE SYNTHESIS: THE INTERACTION BETWEEN SoC AND LoC

While SoC and LoC are related constructs, this review reveals they operate through distinct mechanisms during pandemic stress. LoC appears to function as a more stable, trait-like predisposition that sets the baseline vulnerability or resilience threshold, while SoC operates as a more dynamic, state-like resource that can fluctuate with circumstances. Reviewed studies suggest a hierarchical model where internal LoC facilitates the maintenance of SoC under stress, creating a protective cascade. Conversely, external LoC appears to accelerate SoC erosion when faced with uncontrollable pandemic stressors. This pattern was most evident in included longitudinal studies, where LoC remained relatively stable while SoC varied with restriction severity and pandemic phases.

Also, gender emerged as a moderating factor across both constructs. Women consistently showed higher external LoC and lower SoC, translating to greater vulnerability to anxiety and depression. (Farhane-Medina et al., 2022; Nolen-Hoeksema, 2012) However, the mechanisms appear to differ: for women, external LoC may reflect socialized tendencies toward relational and contextual attribution styles, while lower SoC may result from real disparities in autonomy and resources during the pandemic, including increased caregiving burdens and employment in high-exposure sectors.

## Conclusion

In conclusion, this review highlights the relevance of Sense of Control (SoC) and Internal Locus of Control (LoC) as protective factors against negative psychological effects during the COVID-19 pandemic. The included studies showed that both SoC and LoC significantly influence the way individuals cope with adversity, promoting psychological well-being by strengthening the perception of control over actions, outcomes, and external challenges. Notable, these constructs appear to operate through distinct but complementary mechanisms, with LoC functioning as a stable trait-like foundation that facilitates the maintenance of SoC - a more dynamic, state-like resource - under pandemic stress. These associations suggest that promoting the perception of control mitigates symptoms of depression, anxiety, and pandemic fatigue, by enhancing the use of internal resources to cope with adverse situations.

The consistent gender differences observed across studies, with women demonstrating higher external LoC and lower SoC alongside greater vulnerability to anxiety and depression, indicate that interventions must be gender-sensitive and address both psychological and sociocultural factors. Implementing strategies to strengthen these psychological constructs could be instrumental in reducing the impact of future crises and supporting mental health in times of uncertainty. Such strategies should acknowledge that control perceptions are shaped not only by individual cognitive patterns but also by real disparities in autonomy, resources, and social conditions, particularly affecting women and other vulnerable groups during global health crises.However, it is essential to expand the existing evidence base. The studies reviewed used observational designs, which limits the possibility of establishing causal relationships. To improve understanding of these associations, future studies should adopt experimental and quasi-experimental approaches, capable of evaluating specific instructions and determining the direct impact of strengthening the SoC and internal LoC on mental health.

## Limitations

This study has some limitations that should be considered when interpreting results. Firstly, it is worth mentioning that the inclusion of articles investigating both Locus of Control and Sense of Control represents a possible limitation since although both constructs address perceived control, they have different theoretical and operational bases, which may influence the interpretation of results. For this reason, results and discussion of both constructs were separated during the production of the article to improve clarity and interpretation of findings.

Additionally, contextual factors related to the COVID-19 pandemic pose limitations. Many studies relied on digital data collection methods, primarily online surveys, which, while practical and necessary given pandemic restrictions, introduce potential response biases. Consequently, this may have generated an unrepresentative sample, restricting the generalization of the results, especially concerning the population with less technological familiarity or limited access to digital resources.

A further limitation stems from the observational nature of the studies included in this review. Most of the research relied on cross-sectional designs, which capture data at a single point in time and limit the ability to establish causal relationships between perceived control (SoC and LoC) and mental health outcomes. While correlations suggest meaningful associations, they do not determine whether a stronger sense of control actively mitigates anxiety and depression or whether individuals with lower distress levels naturally perceive more control over their circumstances. Future studies should incorporate longitudinal, experimental, or quasi-experimental designs to better assess causality and the potential long-term benefits of strengthening perceived control.

Lastly, measurement inconsistencies across studies represent another challenge. While this review prioritized studies that used validated scales relevant to SoC and LoC, variations in the specific instruments used and their operational definitions could impact comparability. Some studies may have employed different versions of control-related scales or adapted items for pandemic-related contexts, introducing potential inconsistencies in how constructs were assessed. Future research should work toward standardizing measurement approaches to facilitate more direct comparisons across studies.

## Data Availability

The data sets used and/or analyzed during the current study are available from the corresponding author upon reasonable request.

## Abbreviations

LoC: Locus of Control
SoC: Sense of Control
COVID-19: Coronavirus Disease
SARS-CoV-2: Severe Acute Respiratory Syndrome Coronavirus 2
UAE: United Arab Emirates
DASS-21: Depression, Anxiety, and Stress Scales
BSMAS: Bergen Social Media Addiction Scale
BDI: Beck Depression Inventory
PHR: Experience of Public Health Restrictions
ISCS: Internet Social Capital Scale
HAM-A: Hamilton Anxiety Rating Scale
WLCS: Work Locus of Control Scale
PHQ-4: Patient Health Questionnaire-4
ASEBA: Achenbach System of Empirically Based Assessment
ICI: Internal Control Index
FKK: Questionnaire on Competence and Control Beliefs
PTSD: Post-Traumatic Stress Disorder

## References

[CR1] Bergamo, S. (2024). Embracing uncertainty post-COVID-19 crisis: Insights from young people. Health, Risk & Society, 26(7–8), 317–333. 10.1080/13698575.2024.2412795}

[CR2] Msetfi, R., Kornbrot, D., Halbrook, Y. J., & Senan, S. (2022). Sense of control and depression during public health restrictions and the COVID-19 pandemic. International Journal of Environmental Research and Public Health, 19(21). 10.3390/ijerph192114429} 10.3390/ijerph192114429 PMC9658609 36361309

[CR3] Abeles, R. P. (1991). 14—Sense of Control, Quality of Life, and Frail Older People. Em J. E. Birren, J. C. Rowe, J. E. Lubben, & D. E. Deutchman (Orgs.), The Concept and Measurement of Quality of Life in the Frail Elderly (p. 297–314). Academic Press. 10.1016/B978-0-12-101275-5.50018-1

[CR4] Anderson, C., Turner, A. C., Heath, R. D., & Payne, C. M. (2016). On the meaning of grit…and hope…and fate control…and alienation…and locus of control…and…self-efficacy…and…effort optimism…and…. *The Urban Review,**48*(2), 198–219. 10.1007/s11256-016-0351-3

[CR5] Banai, M., & Reisel, W. D. (2007). The influence of supportive leadership and job characteristics on work alienation: A six-country investigation. *Journal of World Business,**42*(4), 463–476. 10.1016/j.jwb.2007.06.007.

[CR6] Bareeqa, S. B., Ahmed, S. I., Samar, S. S., Yasin, W., Zehra, S., Monese, G. M., & Gouthro, R. V. (2021). Prevalence of depression, anxiety and stress in China during COVID-19 pandemic: A systematic review with meta-analysis. *The International Journal of Psychiatry in Medicine,**56*(4), 210–227. 10.1177/0091217420978005 33243029 10.1177/0091217420978005

[CR7] Benassi, V. A., Sweeney, P. D., & Dufour, C. L. (1988). Is there a relation between locus of control orientation and depression? *Journal of Abnormal Psychology,**97*(3), 357–367. 10.1037/0021-843X.97.3.357 3057032 10.1037//0021-843x.97.3.357

[CR8] Berg, M. B., & Lin, L. (2020). Prevalence and predictors of early COVID-19 behavioral intentions in the United States. *Translational Behavioral Medicine,**10*(4), 843–849. 10.1093/tbm/ibaa085 32893867 10.1093/tbm/ibaa085 PMC7499777

[CR9] Bliznashki, S. (2020). On some objective and subjective aspects of discriminating between correlated and independent patterns and their relationship to locus of control and depression. *Journal of Cognitive Psychology,**32*(3), 323–331. 10.1080/20445911.2020.1738440

[CR10] Bueno-Notivol, J., Gracia-García, P., Olaya, B., Lasheras, I., López-Antón, R., & Santabárbara, J. (2021). Prevalence of depression during the COVID-19 outbreak: A meta-analysis of community-based studies. *International Journal of Clinical and Health Psychology,**21*(1), 100196. 10.1016/j.ijchp.2020.07.007 32904715 10.1016/j.ijchp.2020.07.007 PMC7458054

[CR11] Chak, K., & Leung, L. (2004). Shyness and locus of control as predictors of internet addiction and internet use. *CyberPsychology & Behavior,**7*(5), 559–570. 10.1089/cpb.2004.7.559 15667051 10.1089/cpb.2004.7.559

[CR12] Chamaa, F., Bahmad, H. F., Darwish, B., Kobeissi, J. M., Hoballah, M., Nassif, S. B., Ghandour, Y., Saliba, J.-P., Lawand, N., & Abou-Kheir, W. (2021). Ptsd in the COVID-19 era. *Current Neuropharmacology,**19*(12), 2164–2179. 10.2174/1570159X19666210113152954 33441072 10.2174/1570159X19666210113152954 PMC9185760

[CR13] de Man, A. F., & Devisse, T. (1987). Locus of control, mental ability, self-esteem and alienation. *Social Behavior and Personality: An International Journal,**15*(2), 233–236. 10.2224/sbp.1987.15.2.233

[CR14] Deng, J., Zhou, F., Hou, W., Silver, Z., Wong, C. Y., Chang, O., Drakos, A., Zuo, Q. K., & Huang, E. (2021). The prevalence of depressive symptoms, anxiety symptoms and sleep disturbance in higher education students during the COVID-19 pandemic: A systematic review and meta-analysis. *Psychiatry Research,**301*, 113863. 10.1016/j.psychres.2021.113863 33984824 10.1016/j.psychres.2021.113863 PMC9225824

[CR15] Eatough, E. M., & Spector, P. E. (2014). The role of workplace control in positive health and wellbeing. Em Work and wellbeing, Vol. III (p. 91–109). Wiley Blackwell. 10.1002/9781118539415.wbwell021

[CR16] Eren, F., Kousignian, I., Wallez, S., Melchior, M., & Mary-Krause, M. (2023). Association between individuals’ locus of control and mental health during the COVID-19 pandemic. *Journal of Affective Disorders Reports*. 10.1016/j.jadr.2023.100678

[CR17] Everest, P. (2024). The Relationship Between Covid-19 Social Isolation, Social Anxiety, and Locus of Control. Brescia Psychology Undergraduate Honours Theses. https://ir.lib.uwo.ca/brescia_psych_uht/55

[CR18] Ferreira, M., Rodrigues, J., Pimenta, F., & Patrão, I. (2022). Validation of the pandemic fatigue scale and its COVID-19-related predictors. Psicologia, Saúde & Doença, 23, 1–13. 10.15309/22psd230101

[CR19] Filho, C. R. C., Branco, J. G. de O., Oliveira, T. de Q., Brilhante, A. V. M., Silva, C. A. B. da, & Abdon, A. P. V. (2023). Motivações para o uso do smartphone e tempo de uso por adultos durante a pandemia COVID-19. Medicina (Ribeirão Preto), 56(2), Artigo 2. 10.11606/issn.2176-7262.rmrp.2023.195478

[CR20] Gallagher, M. W., Bentley, K. H., & Barlow, D. H. (2014). Perceived control and vulnerability to anxiety disorders: A meta-analytic review. *Cognitive Therapy and Research,**38*(6), 571–584. 10.1007/s10608-014-9624-x 10.1007/s10608-013-9587-3 PMC3927880 24563563

[CR21] Groth, N., Schnyder, N., Kaess, M., Markovic, A., Rietschel, L., Moser, S., Michel, C., Schultze-Lutter, F., & Schmidt, S. J. (2019). Coping as a mediator between locus of control, competence beliefs, and mental health: A systematic review and structural equation modelling meta-analysis. *Behaviour Research and Therapy,**121*, 103442. 10.1016/j.brat.2019.103442 31430689 10.1016/j.brat.2019.103442

[CR22] Guttmann-Ducke, C., Klinger, S., Ziesche, R., Otzelberger, B., Idzko, M., Ponocny, A., Prantl, S. G., & Ponocny-Seliger, E. (2023). Personality traits and mental distress after COVID-19 testing. Prospective long-term analysis in a Viennese cohort. *Frontiers in Psychiatry,**14*, 1129794–1129794. 10.3389/fpsyt.2023.1129794 36846237 10.3389/fpsyt.2023.1129794 PMC9944018

[CR23] Hong, J. H., Lachman, M. E., Charles, S. T., Chen, Y., Wilson, C. L., Nakamura, J. S., VanderWeele, T. J., & Kim, E. S. (2021). The positive influence of sense of control on physical, behavioral, and psychosocial health in older adults: An outcome-wide approach. *Preventive Medicine,**149*, 106612. 10.1016/j.ypmed.2021.106612 33989673 10.1016/j.ypmed.2021.106612

[CR24] Ko, N.-Y., & Hsu, S.-T. (2005). Informational needs, health locus of control and uncertainty among women hospitalized with gynecological diseases. *Chang Gung Medical Journal,**28*(8), 559–566. 16265846

[CR25] Krampe, H., Danbolt, L. J., Haver, A., Stålsett, G., & Schnell, T. (2021). Locus of control moderates the association of COVID-19 stress and general mental distress: Results of a Norwegian and a German-speaking cross-sectional survey. *BMC Psychiatry,**21*(1), 437–437. 10.1186/s12888-021-03418-5 34488667 10.1186/s12888-021-03418-5 PMC8419811

[CR26] Mahat-Shamir, M., Zychlinski, E., & Kagan, M. (2023). Psychological distress during the COVID-19 pandemic: An integrative perspective. *PLoS ONE,**18*(10), e0293189. 10.1371/journal.pone.0293189 37883473 10.1371/journal.pone.0293189 PMC10602244

[CR27] Mahmoud, A. B., Reisel, W. D., Fuxman, L., & Hack-Polay, D. (2022). Locus of control as a moderator of the effects of COVID-19 perceptions on job insecurity, psychosocial, organisational, and job outcomes for MENA region hospitality employees. *European Management Review,**19*(2), 313–332. 10.1111/emre.12494

[CR28] Manchia, M., Gathier, A. W., Yapici-Eser, H., Schmidt, M. V., de Quervain, D., van Amelsvoort, T., & Vinkers, C. H. (2022). The impact of the prolonged COVID-19 pandemic on stress resilience and mental health: A critical review across waves. *European Neuropsychopharmacology,**55*, 22–83. 10.1016/j.euroneuro.2021.10.864 34818601 10.1016/j.euroneuro.2021.10.864 PMC8554139

[CR29] Maqbali, M. A., Alsayed, A., Hughes, C., Hacker, E., & Dickens, G. L. (2024). Stress, anxiety, depression and sleep disturbance among healthcare professional during the COVID-19 pandemic: An umbrella review of 72 meta-analyses. *PLoS ONE,**19*(5), e0302597. 10.1371/journal.pone.0302597 38722888 10.1371/journal.pone.0302597 PMC11081353

[CR30] Maršanić, V. B., Prijatelj, K., Raguž, A., Kavarić, N., & Flander, G. B. (2023). Predictors of adults’ mental health during initial stage of COVID-19 pandemic in Croatia. *Archives of Psychiatry Research,**59*(2), 197–208. 10.20471/dec.2022.59.02.03

[CR31] Masaeli, N., & Farhadi, H. (2021). Prevalence of internet-based addictive behaviors during COVID-19 pandemic: A systematic review. *Journal of Addictive Diseases,**39*(4), 468–488. 10.1080/10550887.2021.1895962 33749537 10.1080/10550887.2021.1895962

[CR32] Msetfi, R. M., Kornbrot, D. E., & Halbrook, Y. J. (2024). The association between the sense of control and depression during the COVID-19 pandemic: A systematic review and meta-analysis. *Frontiers in Psychiatry*. 10.3389/fpsyt.2024.1323306 38414499 10.3389/fpsyt.2024.1323306 PMC10897004

[CR33] Onyedire, N. G., Ekoh, A. T., Chukwuorji, J. C., & Ifeagwazi, C. M. (2017). Posttraumatic stress disorder (PTSD) symptoms among firefighters: Roles of resilience and locus of control. *Journal of Workplace Behavioral Health,**32*(4), 227–248. 10.1080/15555240.2017.1369885

[CR34] Rotter, J. B. (1966). Generalized expectancies for internal versus external control of reinforcement. *Psychological Monographs: General and Applied,**80*(1), 1–28. 10.1037/h0092976 5340840

[CR35] Ryan, R. M., & Deci, E. L. (2000). Self-determination theory and the facilitation of intrinsic motivation, social development, and well-being. *American Psychologist,**55*(1), 68–78. 10.1037/0003-066x.55.1.68 11392867 10.1037//0003-066x.55.1.68

[CR36] Santomauro, D. F., Herrera, A. M. M., Shadid, J., Zheng, P., Ashbaugh, C., Pigott, D. M., Abbafati, C., Adolph, C., Amlag, J. O., Aravkin, A. Y., Bang-Jensen, B. L., Bertolacci, G. J., Bloom, S. S., Castellano, R., Castro, E., Chakrabarti, S., Chattopadhyay, J., Cogen, R. M., Collins, J. K., … Ferrari, A. J. (2021). Global prevalence and burden of depressive and anxiety disorders in 204 countries and territories in 2020 due to the COVID-19 pandemic. *The Lancet,**398*(10312), 1700–1712. 10.1016/S0140-6736(21)02143-7 10.1016/S0140-6736(21)02143-7 PMC8500697 34634250

[CR37] Santos, L. B., Cunha, L. R. L. da, Santos, Z. C. dos, Silva, A. C. M. M., Silva, P. G. N. da, Medeiros, P. C. B. de, & Medeiros, E. D. de. (2023). Medo da Covid-19 e sofrimento psicológico mediado pela dependência no Smartphone. Revista CPAQV - Centro de Pesquisas Avançadas em Qualidade de Vida, 15(3), Artigo 3. 10.36692/V15N3-27R

[CR38] Schlechter, P., Hellmann, J. H., & Morina, N. (2023). Self-efficacy and locus of control as transdiagnostic factors in Middle Eastern refugees. *European Journal of Psychotraumatology,**14*(1), 2180707. 10.1080/20008066.2023.2180707 37052105 10.1080/20008066.2023.2180707 PMC9987726

[CR39] Shah, A. D., Laternser, C., Tatachar, P., & Duong, P. (2024). The COVID-19 pandemic and its effects on mental health—A before, during, and after comparison using the U.S. Census Bureau’s Household Pulse Survey. *International Journal of Environmental Research and Public Health,**21*(10), 10. 10.3390/ijerph21101306 10.3390/ijerph21101306 PMC11507479 39457279

[CR40] Shin, S., & Lee, E. (2021). Relationships among the internal health locus of control, mental health problems, and subjective well-being of adults in South Korea. *Healthcare (Basel),**9*(11), 11. 10.3390/healthcare9111588 10.3390/healthcare9111588 PMC8620821 34828633

[CR41] Sigurvinsdottir, R., Thorisdottir, I. E., & Gylfason, H. F. (2020). The impact of COVID-19 on mental health: The role of locus on control and internet use. *International Journal of Environmental Research and Public Health*. 10.3390/ijerph17196985 32987750 10.3390/ijerph17196985 PMC7579380

[CR42] Skinner, E. A. (1996). A guide to constructs of control. *Journal of Personality and Social Psychology,**71*(3), 549–570. 10.1037/0022-3514.71.3.549 8831161 10.1037//0022-3514.71.3.549

[CR43] Solomon, Z., Mikulincer, M., & Avitzur, E. (1988). Coping, locus of control, social support, and combat-related posttraumatic stress disorder: A prospective study. *Journal of Personality and Social Psychology,**55*(2), 279–285. 10.1037/0022-3514.55.2.279 3171908 10.1037//0022-3514.55.2.279

[CR44] Sullivan, S. A., Thompson, A., Kounali, D., Lewis, G., & Zammit, S. (2017). The longitudinal association between external locus of control, social cognition and adolescent psychopathology. *Social Psychiatry and Psychiatric Epidemiology,**52*(6), 643–655. 10.1007/s00127-017-1359-z 28271211 10.1007/s00127-017-1359-z PMC5487605

[CR45] Sunde, R. M., Giquira, S., & Aussene, M. M. (2022). Efeitos da pandemia da COVID-19 na saúde mental dos universitários: Caso de estudantes da Universidade Rovuma, Moçambique. Cadernos Ibero-Americanos de Direito Sanitário, 11(2), 88–102. 10.17566/ciads.v11i2.869

[CR46] Vally, Z., Helmy, M., & Fourie, F. (2023). The association between depression and addictive social media use during the COVID-19 pandemic: The mediating role of sense of control. *PLoS ONE,**18*(9), e0291034. 10.1371/journal.pone.0291034 37683017 10.1371/journal.pone.0291034 PMC10490948

[CR47] Whittemore, R., & Knafl, K. (2005). The integrative review: Updated methodology. *Journal of Advanced Nursing,**52*(5), 546–553. 10.1111/j.1365-2648.2005.03621.x 16268861 10.1111/j.1365-2648.2005.03621.x

[CR48] Wu, Q., Zhang, T.-MWang, X., & Zhang, Y. (2024). Pandemic fatigue and depressive symptoms among college students in the COVID-19 context: Indirect effects through sense of control and intolerance of uncertainty. BMC Psychology, 12(1), 21-21. 10.1186/s40359-024-01521-2 10.1186/s40359-024-01521-2 PMC10785367 38212869

[CR49] Yunitri, N., Chu, H., Kang, X. L., Jen, H.-J., Pien, L.-C., Tsai, H.-T., Kamil, A. R., & Chou, K.-R. (2022). Global prevalence and associated risk factors of posttraumatic stress disorder during COVID-19 pandemic: A meta-analysis. *International Journal of Nursing Studies,**126*, 104136. 10.1016/j.ijnurstu.2021.104136 34856503 10.1016/j.ijnurstu.2021.104136 PMC8585564

[CR50] World Health Organization. (2020). Statement on the second meeting of the International Health Regulations (2005) Emergency Committee regarding the outbreak of novel coronavirus (2019-nCoV). https://www.who.int/news/item/30-01-2020-statement-on-the-second-meeting-of-the-international-health-regulations-(2005)-emergency-committee-regarding-the-outbreak-of-novel-coronavirus-(2019-ncov) .

